# Data-driven projections of candidate enhancer-activating SNPs in immune regulation

**DOI:** 10.1186/s12864-025-11374-7

**Published:** 2025-02-26

**Authors:** Markus Hoffmann, Tiago Vaz, Shreeti Chhatrala, Lothar Hennighausen

**Affiliations:** 1 Section of Genetics and Physiology, Digestive and Kidney Diseases, National Institute of Diabetes, National Institutes of Health, Bethesda, MD 20892 USA; 2 Laboratory of Molecular Biology, National Institute of Diabetes and Digestive and Kidney Diseases, National Institutes of Health, Bethesda, MD 20892 USA; 3 Department of Biochemistry and Molecular & Cellular Biology, Georgetown University Medical Center, Washington, D.C. 20007 USA

**Keywords:** Creation of new transcription factor binding sites, GAS motifs, JAK-STAT pathway

## Abstract

**Background:**

Millions of single nucleotide polymorphisms (SNPs) have been identified in humans, but the functionality of almost all SNPs remains unclear. While current research focuses primarily on SNPs altering one amino acid to another one, the majority of SNPs are located in intergenic spaces. Some of these SNPs can be found in candidate cis-regulatory elements (CREs) such as promoters and enhancers, potentially destroying or creating DNA-binding motifs for transcription factors (TFs) and, hence, deregulating the expression of nearby genes. These aspects are understudied due to the sheer number of SNPs and TF binding motifs, making it challenging to identify SNPs that yield phenotypic changes or altered gene expression.

**Results:**

We developed a data-driven computational protocol to prioritize high-potential SNPs informed from former knowledge for experimental validation. We evaluated the protocol by investigating SNPs in CREs in the Janus kinase (JAK) – Signal Transducer and Activator of Transcription (-STAT) signaling pathway, which is activated by a plethora of cytokines and crucial in controlling immune responses and has been implicated in diseases like cancer, autoimmune disorders, and responses to viral infections. The protocol involves scanning the entire human genome (hg38) to pinpoint DNA sequences that deviate by only one nucleotide from the canonical binding sites (TTCnnnGAA) for STAT TFs. We narrowed down from an initial pool of 3,301,512 SNPs across 17,039,967 nearly complete STAT motifs and identified six potential gain-of-function SNPs in regions likely to influence regulation within the JAK-STAT pathway. This selection was guided by publicly available open chromatin and gene expression data and further refined by filtering for proximity to immune response genes and conservation between the mouse and human genomes.

**Conclusion:**

Our findings highlight the value of combining genomic, epigenomic, and cross-species conservation data to effectively narrow down millions of SNPs to a smaller number with a high potential to induce interferon regulation of nearby genes. These SNPs can finally be reviewed manually, laying the groundwork for a more focused and efficient exploration of regulatory SNPs in an experimental setting.

**Supplementary Information:**

The online version contains supplementary material available at 10.1186/s12864-025-11374-7.

## Background

**Fig. 1:**
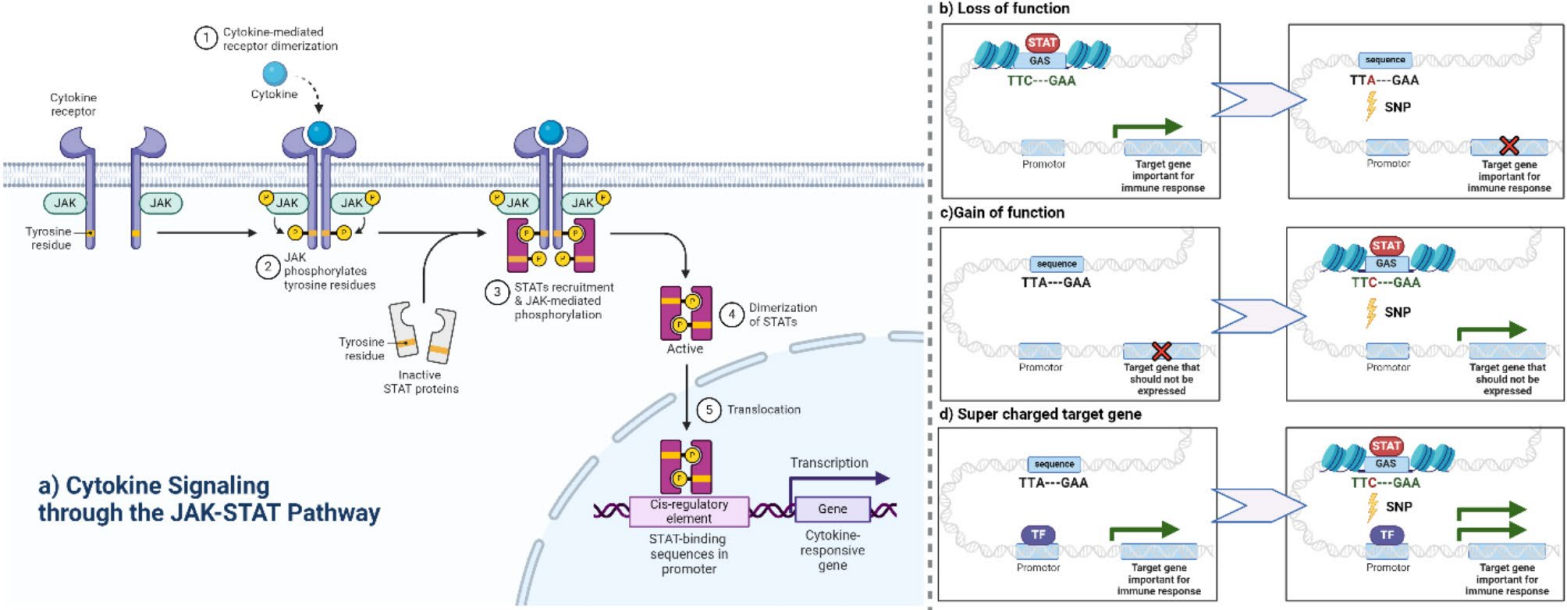
(**a**) Illustration of the exemplary pathway for this study: The JAK-STAT pathway and (**b**-**d**) potential effects of SNPs on cytokine-responsive genes. (**a**) Illustrates the JAK-STAT pathway. Upon cytokine binding, transmembrane receptors dimerize, and JAKs are recruited, which subsequently results in JAK autophosphorylation and the phosphorylation of specific tyrosine residues on both the receptor and the STAT proteins that have been recruited to the receptors. Phosphorylated STATs dimerize, translocate to the nucleus, and bind to GAS motifs with the canonical TTCnnnGAA sequence, which leads to the transcription of target genes involved in immune responses. (**b**) A SNP disrupts the regular GAS motif, preventing STAT transcription factors from binding to the genomic site. As a result, an immune-related gene normally activated by interferons no longer responds to this stimulus, leading to a potential loss of function (LOF) in the immune response. (**c**) A SNP creates a new GAS motif where none existed before. This allows STAT proteins to bind and drive the expression of a gene that should remain off, leading to unintended gain of function (GOF). (**d**) A SNP strengthens the GAS motif or adds additional regulatory factors, producing heightened gene expression. This results in a “supercharged” effect on the immune-related gene

Millions of single nucleotide polymorphisms (SNPs) have been identified in human populations [1] and cataloged in disease databases such as COSMIC [2, 3] and more general databases such as dbSNP [4] and *All of Us* [5]. SNPs can have impacts on gene function (e.g., through mutating the codon of one amino acid into another, resulting in protein variants) and regulation (e.g., by enhancing or creating *de novo* transcription factor binding site (TFBS) or rendering an existing TFBS less effective or destroying them) [6, 7]. While current research focuses primarily on SNPs within the coding regions of genes, most SNPs are located in the intergenic regions and could potentially cause deregulation by altering cis-regulatory elements (CREs) [8]. Deregulation of important target genes in crucial pathways such as the Janus kinase-signal transducer and activator of transcription (JAK-STAT) pathway can be fatal for the survival of the organism [9]. The activation of the JAK-STAT pathway is critical in the immune system [10], leading to the regulation of immune genes, which play a significant role in the body’s response to viral infections [11, 12], autoimmune diseases [13], cancer [14], and a plethora of other conditions [15, 16]. Following cytokine signaling, the JAK-STAT pathway facilitates the phosphorylation and dimerization of STAT transcription factors (TFs), which subsequently translocate to the nucleus to bind gamma-interferon-activated sites (GAS) motifs in CREs such as promoters and enhancers [1] and regulate key immune genes (Fig. 1a [17]). The successful and targeted binding of the STAT TF family to GAS motifs is crucial in regulating the expression levels of immune genes, and expression levels can be significantly disturbed or enhanced by SNPs in the CREs of the genes. Such SNPs in CREs can either destroy/disrupt/enhance existing GAS motifs or create new ones, leading to loss-of-function (LOF), gain-of-function (GOF), or super-charged target gene scenarios (Fig. 1b-d; [18]). LOF, GOF, or super-charged target genes in the JAK-STAT pathway can lead to a deregulated immune system and, ultimately, to disease [19]. Hence, investigating SNPs that could deregulate such critical pathways is crucial for advancing medical science and, eventually, the potential identification of biomarkers and therapeutic targets for the future development of targeted therapies for individuals that show different behaviors in cytokine signaling [7].

In recent years, several computational and experimental findings about SNPs destroying or rendering TFBS less effective have been published [20–22]. On the computational side, most studies only focus on statistical associations without classifying SNPs into regulatory/non-regulatory SNPs and without validating these findings in *in vitro or in vivo* experiments [23–25]. However, the literature is more limited in studies experimentally investigating SNPs that enhance TFBS due to the complexity of the many possibilities of the motif where the TF can bind to the DNA (i.e., many TFs can bind to very similar and overlapping sequences; [26, 27]). Ultimately, the literature contains only a small number of studies focused on SNPs that directly result in the creation of a TFBS in promoters or enhancers (see Suppl. Table 1 and Suppl. Text 1 for a summary of publications where an increase in activity or a creation of a TFBS was shown [28–43]).

We believe the limited number of publications performing a stable insertion of a SNP to create or enhance a regulatory element (five out of the 16 papers in Suppl. Table 1) compared to other methods (nine luciferase reporter assays and two with transient transfections, other than luciferase reporter assays out of 16 papers in Suppl. Table 1) is likely due to the challenging nature and logistics of such projects [44]. Given that only a few of all potential motifs are bound, the likelihood of identifying a SNP that creates a functional binding site, which also regulates a target gene, is low, as demonstrated by Zhu et *al.* [44]. As a result, most researchers prefer using luciferase, avoiding the complexity of stably introducing a SNP into the genome that enhances or creates a TFBS in a mouse model or cell line. With all of these considerations in mind: this presents a long-term and costly project for researchers that might never be published, and we believe this is why most of these studies are computationally inspired or hypothesized as part of a manuscript but are never or only partially investigated in vitro or in vivo.

**Fig. 2:**
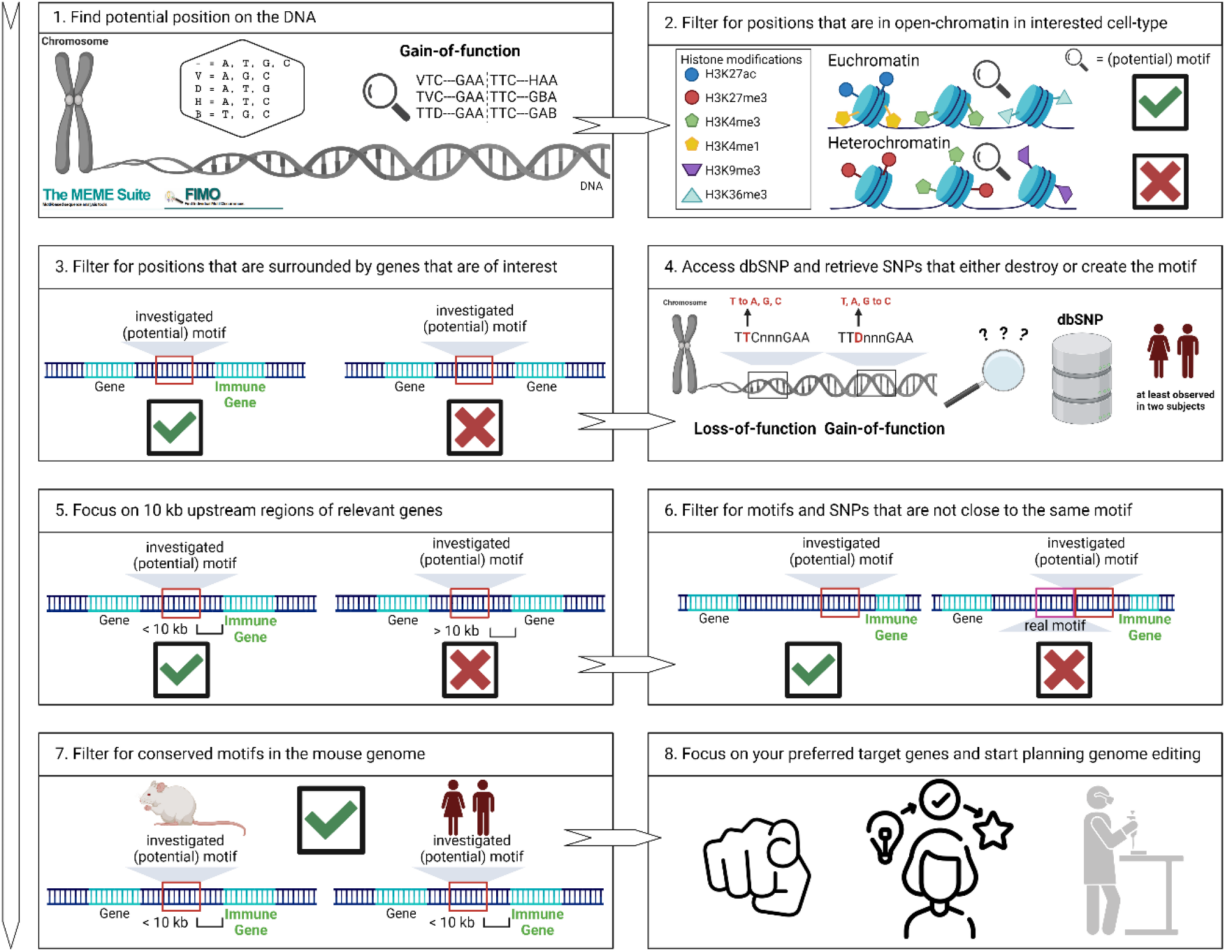
Computational protocol to find SNPs that potentially create a GAS motif and how to prioritize them. (**1**) Identification of potential positions on the genomic DNA: We used the motif-search tool FIMO by the MEME Suite to locate sequences that resemble gain-of-function (GOF) motifs of interest. These candidate sites may have the potential to alter gene expression if mutated. (**2**) Filter for open-chromatin regions in the desired cell type: Narrow down the candidate sites to those found in accessible regions, as indicated by specific histone modifications (e.g., H3K27ac, H3K4me3). Sites in regions of condensed chromatin are less likely to be functionally relevant. (**3**) Focus on candidate sites near genes of interest: Retain only the motifs in the vicinity of genes of interest (e.g., immune-related genes). Discard motifs that lie near genes irrelevant to the research question. (**4**) Check dbSNP for variants that create or disrupt the motif: Confirm that investigated SNPs are documented in databases like dbSNP and appear in at least two individuals to avoid SNPs from sequencing errors. (**5**) Limit the search to 10 kb upstream of Transcriptional Start Sites (TSS): Focus on sequences within 10 kilobases upstream of the gene’s transcription start site. Variants in this enhancer and promoter-proximal region are more likely to affect gene expression. (**6**) Exclude motifs and SNPs that cluster too closely: Remove any candidate motifs or SNPs that are very close to each other or overlap an existing, well-defined motif. This prevents conflicting readouts in downstream analyses. (**7**) Analyze conservation in the mouse genome: Retain motifs conserved in mice, as conservation often indicates functional importance. Non-conserved motifs may be less likely to have a regulatory role. Popular tools like AlphaFold [65] often use conservation as one factor in their decision. (**8**) Choose final target genes and plan editing: With the refined list of candidate sites, identify your top targets and design genome editing strategies (e.g., CRISPR-based approaches) to study or modify these variants in the lab

In this manuscript, we present an adjustable data-driven computational protocol (Fig. 2) designed to prioritize variants capable of creating motifs for essential pathways. We focus on the JAK-STAT pathway, where GAS motifs are of the utmost importance. One defining feature of STAT transcription factors is the requirement of a palindromic DNA binding motif containing six fixed nucleotides (TTCnnnGAA). We identify GAS motifs that are in areas of interest, leveraging the distinct clarity of the GAS motif’s sequence [45]. We intend to inspire more research in which SNPs that create *de novo* TFBS are investigated. The protocol incorporates a conception of how to identify potential sequences genome-wide where a single SNP could establish a TFBS, along with a strategy to refine the selection of sequences to those most plausible for investigation based on the researcher’s expertise and extent of information available in the field. This work aims to inspire bioinformaticians and experimental researchers to collaborate on such crucial studies by initially pinpointing potential candidates through in silico analysis and validating these variants through in vitro experiments and, ideally, in vivo investigations.

## Results and discussion

**Fig. 3:**
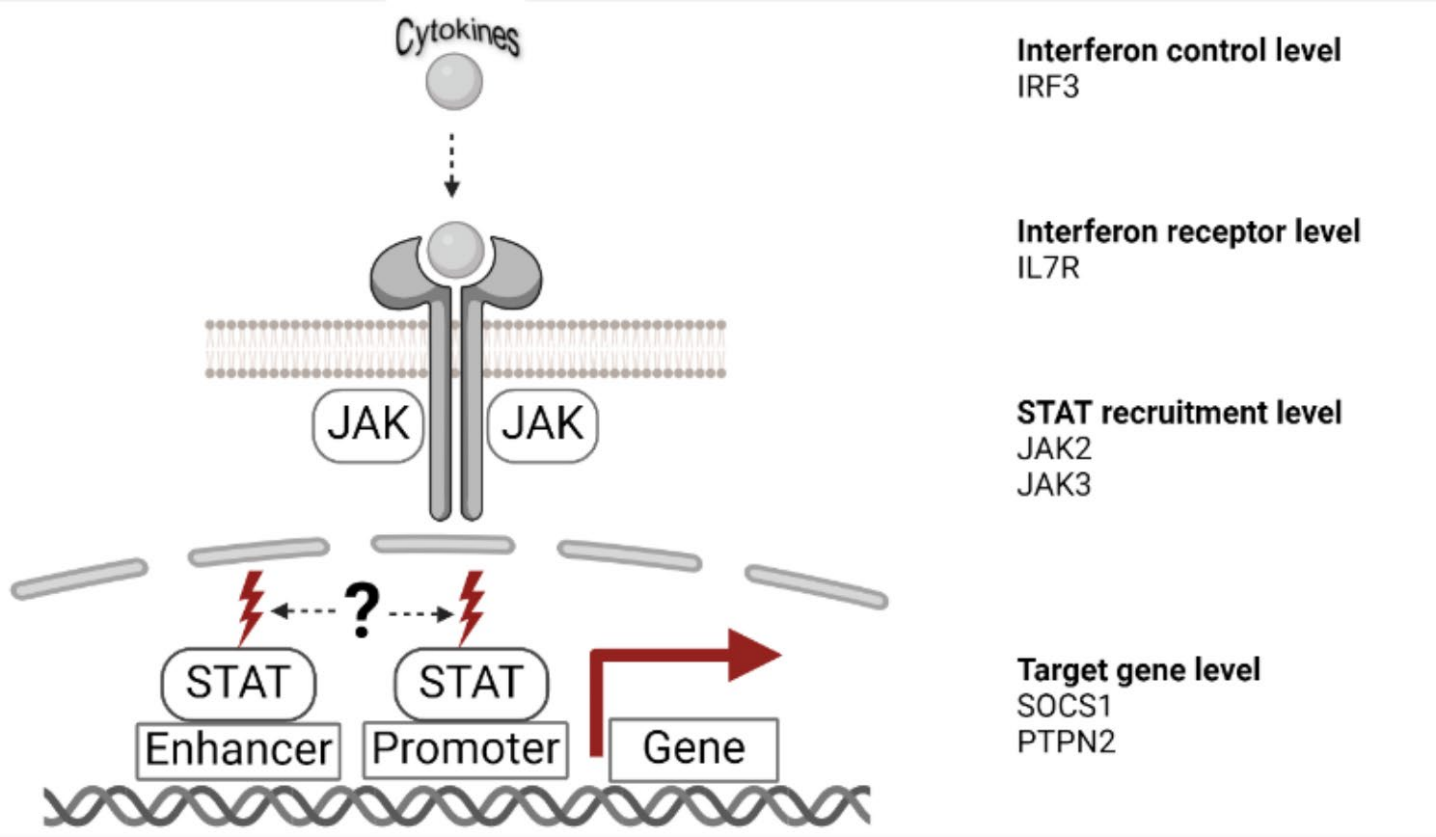
SNPs in enhancers of IRF3, IL7R, JAK2, JAK3, SOCS1, and PTPN2 were further investigated since they represent four key regulatory nodes in the JAK-STAT pathway. At the “interferon control level,” IRF3 helps regulate interferon production. The “interleukin receptor level” is represented by IL7R, where receptor engagement initiates JAK activation. The “STAT recruitment level” involves JAK2 and JAK3 phosphorylating and recruiting STAT proteins to enhancers or promoters. Finally, at the “target gene level,” STAT-driven transcription factors modulate genes such as SOCS1 and PTPN2, which help fine-tune immune signaling

In our protocol (Fig. 2), we initially utilize the computational method FIMO from the MEME suite (see Materials and Methods for parameters) [46] to conduct a comprehensive search across the genome for nearly complete GAS motifs (ncGAS; i.e., where the motif is missing only one nucleotide and can be converted to an intact GAS motif by a single SNP, see Table 1). FIMO found 17,039,967 potential ncGAS motifs across the human genome, and 3,301,512 SNPs (Suppl. Table 2) could be identified in those locations according to dbSNP [4]. In a second step, to ensure the biological relevance of these motifs, we filtered our findings based on their presence within open-chromatin regions, as predicted by active histone modification (H3K27ac) ChIP-seq data, thus prioritizing areas accessible for transcription factor binding (see Materials and Methods for details on the analysis) which result in 50,265 SNPs for PBMC and T-Cells. Next, we focused only on sequence positions that had at least one gene of interest as their neighbor upstream or downstream (i.e., known immune genes by using the Gene Ontology (GO) database [47]; see Materials and Methods for details). This filtering step left 16,017 SNPs for further investigation. We achieved additional refinement by focusing on motifs located within 10 kilobase (kb) upstream regions of transcriptional start sites (TSS) of genes, known for harboring key regulatory elements, leaving 4,391 SNPs for further analysis; [48]. Next, we integrated data from dbSNP [4], which we use to identify specific SNPs with the potential to create intact GAS motifs and only select the ones that were found in at least two independent human subjects to avoid sequencing errors (416 SNPs left). As a next step in our selection process, we wanted to ensure that these motifs and SNPs were sufficiently spaced (i.e., 200 bps from existing GAS motifs) to avoid mutual interference, thus preserving their potential regulatory impact (273 SNPs left). To prioritize SNPs, we examined if we could identify the same type of motif in the mouse genome 10 kb upstream of the same target genes (mm10), focusing on variants that could be introduced into a mouse model (214 SNPs left). Since, most likely, there will still be too many candidates for a feasible mouse project, we emphasized that one could now screen the candidates one by one and choose the ones that the researcher or a collaborator has the most experience and interest. We especially focused on the JAK-STAT pathway, so we further investigate 30 targets out of the 214 SNPs, none of which are reported in ClinVar [49–51] (Suppl. Table 3). This highlights the importance of this work and investing time into exploring such SNPs that could disrupt relevant pathways further. Upon closer investigation, we pick six SNPs (rs560898780, rs1257658099, rs370669851, rs571421696, rs910130021, rs138606888) in enhancers upstream of the genes IRF3, IL7R, JAK2, JAK3, SOCS1, and PTPN2 on several levels of the JAK-STAT pathway to investigate if our protocol results in feasible candidates to conduct gene-editing experiments (Fig. 3).

**Table 1:** The sequence of the GAS motif and assigned names for a potential almost GAS motif with one mutation

Motif	Name
**TTC—GAA**	**GAS motif**
**VTC—GAA**	**T1 GAS motif**
**TVC—GAA**	**T2 GAS motif**
**TTD—GAA**	**C GAS motif**
**TTC—HAA**	**G GAS motif**
**TTC—GBA**	**A1 GAS motif**
**TTC—GAB**	**A2 GAS motif**
V = A, G, C	D = A, T, G
H = A, T, C	B = T, G, C

We selected SNPs in enhancers of these six target genes because of their distinct role on different layers of the JAK-STAT pathway (Fig. 3): (i) regulation of interferons (IRF3), (ii) receptor of interferons (IL7R), (iii) recruitment and phosphorylation of the STAT TF family (JAK2 and JAK3), and (iv) target genes of the JAK-STAT pathway with a negative feedback loop toward the JAK-STAT pathway (SOCS1 and PTPN2). A SNP creating a GAS motif for STAT binding in the IRF3 – a regulator for cytokine expression - enhancer could lead to a hyperactivating immune gene expression and potentially causing autoimmunity through aberrant JAK-STAT pathway activation [52]. Similarly, IL7R, which is essential for T and B cell development, could see enhanced IL-7 signaling due to upregulation from a SNP-induced GAS motif in its enhancer, hypothetically disrupting lymphocyte homeostasis and possibly leading to lymphoproliferative disorders [53]. JAK2, responsible for phosphorylating STAT proteins and activating them to regulate gene expression, may be more abundant in the cell by a SNP in its enhancer, creating a GAS motif [54]. In the case of JAK3, which selectively transduces signals for cytokine receptors involved in lymphocyte development, an upregulation resulting from a GAS motif-enhancing SNP could result in exaggerated immune responses and autoimmune diseases due to higher activation of the JAK-STAT pathway [55]. PTPN2 acts as a negative regulator by dephosphorylating JAKs, and its upregulation through a SNP-induced GAS motif could lead to disruption of this negative feedback, prolonging pathway activation and inflammatory disease progression [56]. On the other hand, SOCS1, a direct inhibitor of JAK kinase activity, could result in a dysregulated JAK-STAT signaling balance from upregulation driven by a SNP-created GAS motif in its enhancer, potentially leading to immune suppression or resistance to cytokine signaling [57].

**Fig. 4:**
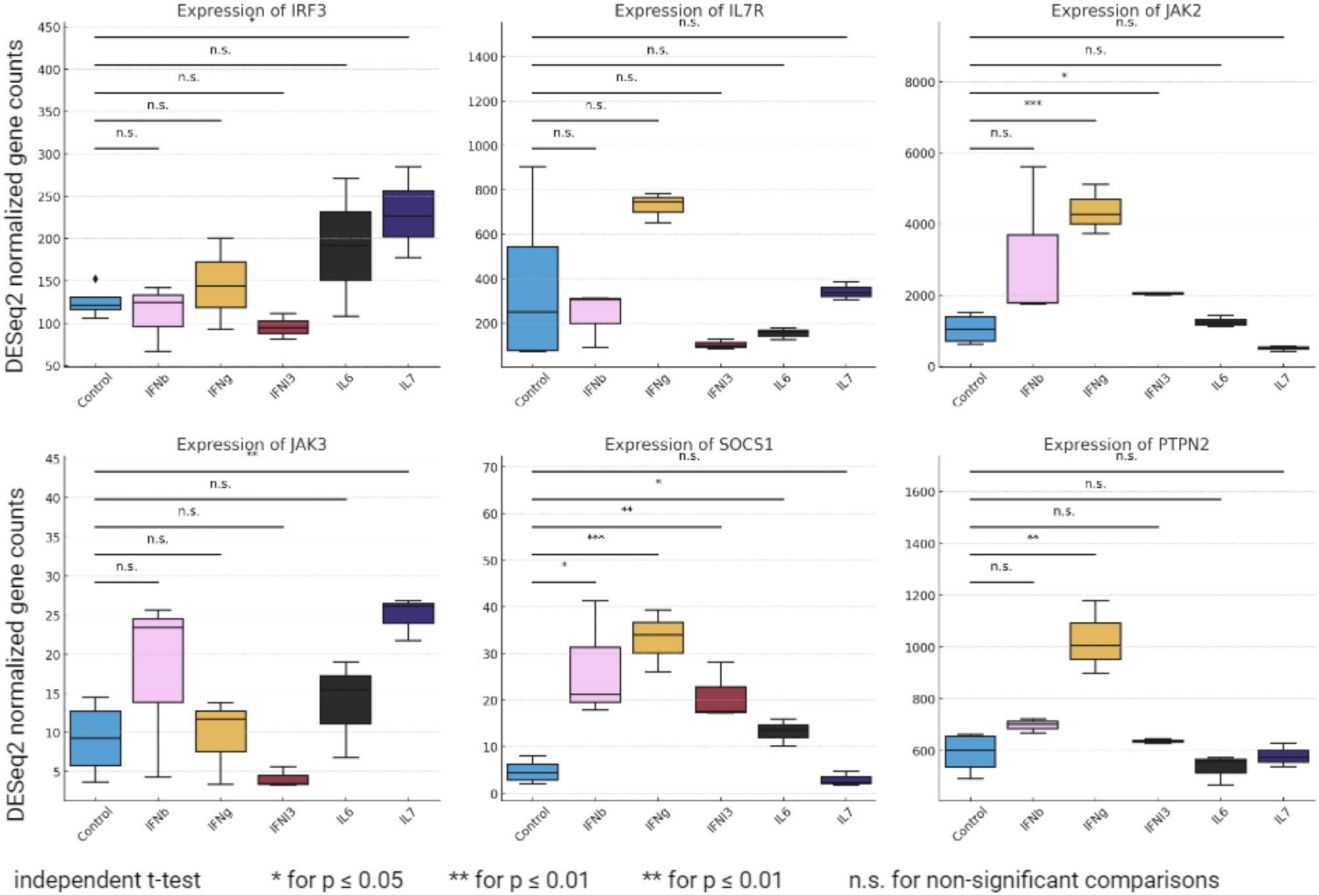
Expression of key genes in the JAK-STAT pathway under different cytokine stimulation conditions. DESeq2 normalized gene counts are presented for IRF3, IL7R, JAK2, JAK3, SOCS1, and PTPN2 across control, IFNα, IFNβ, IFNγ, IL-6, and IL-7 stimulations. Independent t-tests were performed to compare expression levels, with significance indicated as follows: *p ≤ 0.05, **p ≤ 0.01, and n.s. for non-significant comparisons

Next, we investigated if an experimental design of a genome editing approach to evaluate the impact of the selected SNPs was feasible using in vivo animal models and cell line models. Both approaches require precise gene editing to avoid off-target modifications and insertions of transgenes or expression vectors that could lead to insertional mutagenesis or other issues that may compromise the results [58]. In vivo experiments are the most informative as they allow the investigation of the role of the SNPs during development and their impact on specific tissues and on the organism as a whole. Alternatively, cell models are better suited to gather preliminary data, namely to evaluate the cellular and molecular impact of the SNPs and validate the genome editing strategy. One of the first steps in conceptualizing an experimental design for genome editing involves selecting the appropriate cells or animals. When considering in vivo experiments, mouse models are frequently the preferred choice due to their lower handling and costs compared to other animal models [59]. However, using mouse models still comes with significant costs and extended project timelines, often stretching 3–4 years to obtain meaningful results. Given the high costs and long duration associated with mouse models, it is responsible to first seek preliminary evidence in less complex systems like primary cells or cell lines [60]. Primary cells, derived directly from living tissue, offer a non-duplicated genome and an absence of genetic changes that accompany immortalization when generating cell lines, thus enhancing the biological relevance of the results [61]. However, their handling is often more difficult and more expensive, alongside the complication of their limited number of doubling times in vitro [62]. Cell lines, in contrast, provide a more accessible and manageable alternative. These cells can proliferate indefinitely under the right conditions, offering a stable and reproducible system for genetic manipulation (Suppl. Text 2, Suppl. Figures 1,2,3) [63]. To assess whether pinpointed SNPs’ creation of GAS motifs could lead to de novo activation or enhance existing gene expression, we analyzed publicly available RNA-seq data under various cytokine stimulation conditions. Lee et al. [11] investigated the effects of interferon stimulations on primary cells derived from human lungs. The resulting expression patterns of key genes involved in the JAK-STAT signaling pathway were compared across different stimulation conditions, including IFNα, IFNβ, IFNγ, IL-6, and IL-7. Notably, the genes JAK2, SOCS1, and PTPN2 exhibited significant differential expression, indicating potential enhancement of the JAK-STAT pathway through the modification of GAS motifs (Fig. 4). We further checked if we found acetylation at the identified SNPs in independent ChIP-seq data from IL4-stimulated PBMC cells [64]. We confirmed the accessible CREs, similar to the ChIP-seq data used in our protocol (Suppl. Figure 4).

### Considerations and current challenges with SNP databases

The data utilized in this study was derived from experiments designed with different hypotheses in mind, which may not align perfectly with our specific objectives - despite a thorough investigation into data quality (see Materials and Methods). A significant limitation of our approach is the focus on SNPs within 10 kb upstream of genes, based on the assumption that this region is rich in enhancers and regulatory elements. However, this could lead to overlooking significant candidates located downstream of genes. Moreover, excluding distant CREs that can be as far as 50 kb away limits our study’s scope (Suppl. Text 3).

A key limitation of current SNP repositories is that they only provide sequence information without accompanying patient materials or primary cells. This makes it impossible to carry out direct functional experiments, such as Sanger sequencing or transcription factor (TF) ChIP-seq, on specific SNPs of interest. For instance, verifying a novel GAS motif in an enhancer region would require access to cells from an individual carrying the SNP, but such data or materials are not available in dbSNP or similar resources. Conducting such studies further depends on identifying and recruiting multiple donors with the exact SNP, which is often not feasible due to privacy restrictions.

Another challenge arises if the transcription factor motif in question is relatively short or “wobbly”—as may be the case for certain TF families (e.g., AB1 or ESR1) —it becomes even more challenging to interpret the biological impact of the SNP. By contrast, SNPs that alter more defined motifs (e.g., NFIB sites) are easier to evaluate.

Finally, the positional context of SNPs can create uncertainty. Some non-coding SNPs lie between genes or within the overlapping regions of multiple genes, so it is not always clear which gene(s) may be influenced. There is evidence that there can be regulatory elements, sometimes they are called super-enhancers, that can regulate two genes simultaneously, as shown by Lee et *al.* (2023) [66]. Without experimental data or more detailed expression profiles, the link between such SNPs and downstream functional changes remains purely speculative. Strengthening SNP databases through deeper annotation, integrating patient-derived samples, and better access to transcriptomic and epigenomic data would help overcome these limitations and enable more robust functional validation of candidate regulatory variants.

## Conclusion

Despite limitations inherent to existing genomic databases, our computational protocol identified potential ‘de novo’ generated TF binding sites, potentially generating enhancers that could impact immune regulation. Our data-driven protocol provides targets for further investigation in the laboratory, which will require primary cells from human patients, and demonstrates the feasibility of transitioning from in silico analyses to experimental validation. With this approach, we highlight the challenges and opportunities of collaboration between experimental biologists and computational biologists. Bridging those two crucial fields will provide clear benefits to the scientific community. Our findings highlight the value of combining genomic, epigenomic, and cross-species conservation data to effectively narrow down millions of SNPs to a smaller number with high regulatory potential that can finally be reviewed manually. This approach sets the stage for a more targeted and efficient investigation of regulatory SNPs in experimental studies.

## Materials and methods

### Materials

We used all SNPs from dbSNP version 155. We further used the sequence of hg38 to find GAS motifs or identify potential almost GAS motifs. We used the sequence of mm10 to investigate for potential conserved motifs in mice. We used a plethora of datasets from the database Gene Expression Omnibus (GEO) accessible at https://www.ncbi.nlm.nih.gov/geo/ with the following GSE (dataset) and GSM (sample) identifiers. We used the histone modification (H3K27ac) ChIP-seq data from GSE212588 [67] and GSE116695 (GSM6538036, GSM6538038, GSM3258553, GSM3258554, and GSM3258556) [68]. Transcription factor ChIP-seq data: STAT1: GSE31477 (GSM935612) [69]; STAT3: GSE117164 (GSM3272738) [70]; STAT5: GSE43119 (GSM1056920) [71]. FASTQ files were processed with the Galaxy web interface (https://usegalaxy.org/) to map sequences of ChIP-seq (Bowtie2) and were converted from BAM to bigWig using the bedtools function in Galaxy (both tools were executed using default parameters set by Galaxy). RNA-seq data: GSE189997 (GSE189997_genecount_table.tsv.gz) [72], GSE215771 (GSM6638919, GSM6638920, GSM6638926, GSM6638927, GSM6638933, GSM6638934) [73], GSE178640 (GSM5395133, GSM5395134, GSM5395135, GSM5395136, GSM5395137, GSM5395138) [74], GSE35267 (GSM864771, GSM864772, GSM864773, GSM864753, GSM864754, GSM864755) [75], GSE46599 (GSM1133044, GSM1133045, GSM1133046, GSM1133047) [76], GSE161664 [11]. RNA-seq data was normalized using the DESeq2 method using standard parameters. For detailed sample numbers of RNA-seq data per condition/stimulation, we refer to Suppl. Table 4. Independent ChIP-seq data from IL4-stimulated PBMC were obtained from: GSE100889 (GSM2695648, GSM2695649) [64]. We checked the quality of the ChIP-seq data by verifying known peaks (e.g., near BCL6, CISH).

### Methods

#### FIMO by MEME suite

FIMO is a tool within the MEME Suite designed for scanning DNA or protein sequences for occurrences of motifs. To utilize FIMO to find a defined sequence genome-wide, you first need a motif of interest, which can be represented in various formats, including the Position Weight Matrix (PWM). For the study on the GAS motifs being destroyed by SNPs, we used the motifs in Suppl. Textbox 1 for the canonical and non-canonical motif and we used the motifs in Suppl. Textbox 2 for the T1 GAS, T2 GAS, C GAS, G GAS, A1 GAS, and A2 GAS motifs. We further used the parameters **“--thresh 0.05 --no-qvalue --max-stored-scores 900000000** ” and ran the tool on each chromosome and motif individually to capture all potential motifs. FIMO was run locally due to runtime and memory issues on Galaxy for the genome-wide search.

#### Preprocessing of open-chromatin files of investigated cell types (e.g., H3K27ac) for the protocol

FASTQ files were uploaded to https://usegalaxy.org/. Bowtie2 with default parameters and the hg38 genome was used to map the reads to the genes and retrieve gene expression. The command line was:

set -o | grep -q pipefail && set -o pipefail; ln -s ‘/data’ input_f.fastq.gz && bowtie2 -p ${GALAXY_SLOTS:-4} -x ‘/cvmfs/data.galaxyproject.org/byhand/hg38/hg38full/bowtie2_index/hg38full’ -U ‘input_f.fastq.gz’ | samtools sort --no-PG -@${GALAXY_SLOTS:-2} -T “${TMPDIR:-.}” -O bam -o ‘data.dat’.

The BAM files were then transformed into bed and bigWig files by bedtools v. 2.29.2 and ucsc-bedgraphtobigwig v. 377 at https://usegalaxy.org/.

bedtools genomecov -bg -split -ibam ‘data.dat’ | LC_COLLATE   =   C sort -k1,1 -k2,2n   >   temp.bg && bedGraphToBigWig temp.bg ‘/cvmfs/data.galaxyproject.org/managed/len/ucsc/hg38.len’ ‘data.dat’.

We used the command line **awk ‘$2**   **>**   **20’ your_data_file.bed** to detect peaks where we find an acetylation signal of 20 or higher [66]. We then used the bedfile created by the awk command and the bedfiles created by FIMO in the bedtools window with a window size of 200 since this is the approximal length for the peak–valley–peak model where TF binding, and the acetylation levels would be decreases [77, 78].

#### Retrieval of a list of immune genes

To retrieve a list of immune genes, we utilized the Gene Ontology (GO) database [47] and used all genes that were in or below the following GO Terms in the acyclic graph: Immune System: GO:0002376, Immune Response: GO:0006955, Cytokine Signaling: GO:0019221, Interferon Signaling: GO:0060333, Interleukin Signaling: GO:0070102, JAK-STAT Pathway: GO:0007259. This resulted in a total of 2,045 genes.

#### Remove all potential GAS motifs for gain-of-function study from investigation

We used all genome-wide found GAS motifs by FIMO and removed all potential GAS motifs (e.g., T1 GAS, T2 GAS,… A2 GAS) from the analysis, which fell into a window of 200 bp [77, 78] in the surroundings (using the bedtools window function). We remove such potential GAS motifs since, from the space requirement, no second TF could bind there [77, 78].

## Electronic supplementary material

Below is the link to the electronic supplementary material.

Supplementary Material 1

## Data Availability

The Python code underlying the protocols execution for investigating GAS motifs is available at: https://github.com/Firestar93/GAS_motifs dbSNP version 155 was used from https://hgdownload.soe.ucsc.edu/gbdb/hs1/dbSNP155/. The sequence of hg38 was used to find GAS motifs or identify almost all GAS motifs from https://hgdownload2.soe.ucsc.edu/goldenPath/hg38/chromosomes/. The sequence of mm10 was used to investigate for (potential motifs) https://hgdownload2.soe.ucsc.edu/goldenPath/mm10/chromosomes/. We used a plethora of datasets from the database Gene Expression Omnibus (GEO), accessible at https://www.ncbi.nlm.nih.gov/geo/ with the following GSE identifiers. Sample identifiers (GSM) can be found in the Materials and Methods section. Histone modification ChIP-seq data: GSE212588; GSE116695; independent open chromatin check: GSE100889. Transcription factor ChIP-seq data: STAT1: GSE31477; STAT3: GSE117164; STAT5: GSE43119RNA-seq data: GSE189997, GSE215771, GSE178640, GSE35267, GSE46599. All results and selected inputs of the different steps for reproducibility can be downloaded here: https://doi.org/10.6084/m9.figshare.26103079.
