# Supplementary material for: Data-driven projections of candidate enhancer-activating SNPs in immune regulation
Source: BMC Genomics. 2025 Feb 26;26:197. doi: 10.1186/s12864-025-11374-7 (PMC11863423; doi:10.1186/s12864-025-11374-7)
Supplement: Supplementary file 1 — Supplementary Material 1 [file 12864_2025_11374_MOESM1_ESM.docx]

***Supplementary Materials for* Data-driven projections of candidate enhancer-activating SNPs in immune regulation**

Markus Hoffmann^1,*^, Tiago Vaz^2^, Shreeti Chhatrala^1,3^, and Lothar Hennighausen^1^

^1^ Laboratory of Genetics and Physiology, National Institute of Diabetes, Digestive and Kidney Diseases, National Institutes of Health, Bethesda, MD, USA
^2^ Laboratory of Molecular Biology, National Institute of Diabetes and Digestive and Kidney Diseases, National Institutes of Health, Bethesda, MD, 20892, USA
^3^ Department of Biochemistry and Molecular & Cellular Biology, Georgetown University Medical Center
* corresponding author: [markus.hoffmann@nih.gov](mailto:markus.hoffmann@nih.gov)

**ABSTRACT**

**Background:** Millions of single nucleotide polymorphisms (SNPs) have been identified in humans, but the functionality of almost all SNPs remains unclear. While current research focuses primarily on SNPs altering one amino acid to another one, the majority of SNPs are located in intergenic spaces. Some of these SNPs can be found in candidate cis-regulatory elements (CREs) such as promoters and enhancers, potentially destroying or creating DNA-binding motifs for transcription factors (TFs) and, hence, dysregulating expression of nearby genes. These aspects are understudied due to the sheer number of SNPs and TF binding motifs, making it challenging to identify SNPs that yield phenotypic changes or altered gene expression.

**Results:** We developed a data-driven computational protocol to prioritize high-potential SNPs educated from former knowledge for experimental validation. We evaluated the protocol by investigating SNPs in CREs in the JAK-STAT signaling pathway, which is crucial in controlling immune responses and has been implicated in diseases like cancer, autoimmune disorders, and responses to viral infections. The protocol involves scanning the entire human genome (hg38) to pinpoint DNA sequences that deviate by only one nucleotide from binding sites for STAT TFs. We identified six SNPs in regions likely to influence regulation within the JAK-STAT pathway, narrowing down from an initial pool of 3,301,512 SNPs across 17,039,967 nearly complete STAT motifs. This selection was guided by publicly available open chromatin and gene expression data and further refined by filtering for proximity to immune response genes and conservation between the mouse and human genomes.

**Conclusion:** Our findings highlight the value of combining genomic, epigenomic, and cross-species conservation data to effectively narrow down millions of SNPs to a smaller number with high regulatory potential that can finally be reviewed manually, laying the groundwork for a more focused and efficient exploration of regulatory SNPs in an experimental setting.

**KEYWORDS**

Creation of New Transcription Factor Binding Sites; GAS motifs; JAK-STAT pathway

**Supplementary Figure 1: Gene editing design for the six targets prioritized by the computational protocol**

**
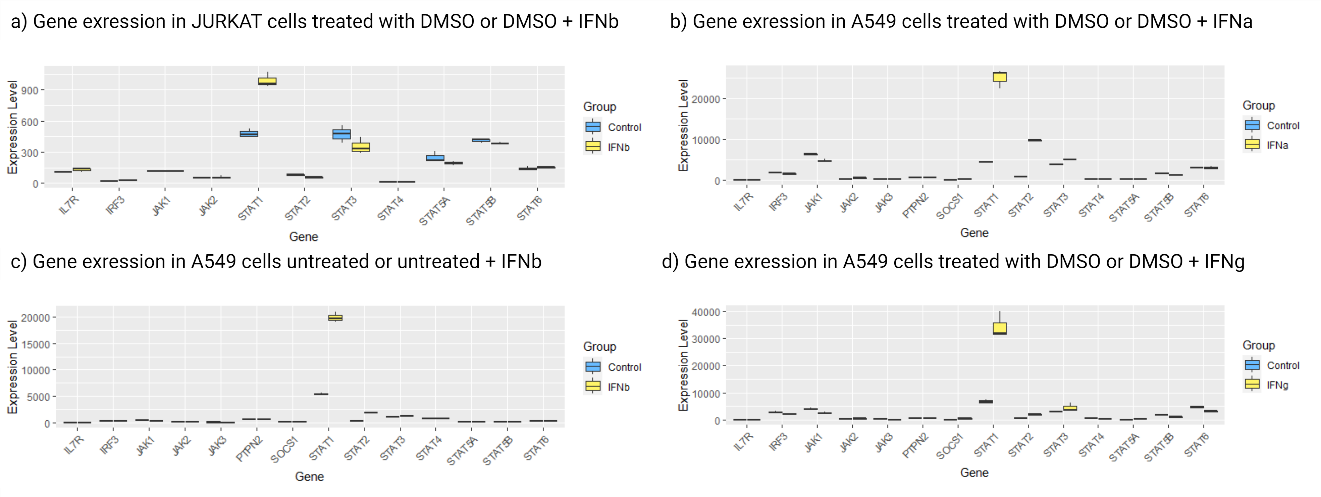
**

Supplementary Figure 1: After a thorough investigation, we identified one JURKAT study and three A549 studies with untreated and interferon-treated (IFNa, IFNb, and IFNg) samples. The RNA-seq or NanoString data shows that all the main components and our target genes are expressed in both cell lines. Moreover, our target genes do not seem to be under the control of the JAK-STAT pathway and interferon response. Additionally, STAT1 expression is increased on stimulated conditions, suggesting that the JAK-STAT pathway can be activated in those cell lines. Changes of expression levels of important JAK-STAT genes and the target genes in JURKAT and A549 cells stimulated and stimulated with IFNa, IFNb, and IFNg to investigate which genes are under control of the JAK-STAT pathway and if the cell line can activate the JAK-STAT pathway.

**Supplementary Figure 2: Homology-directed repair**

**
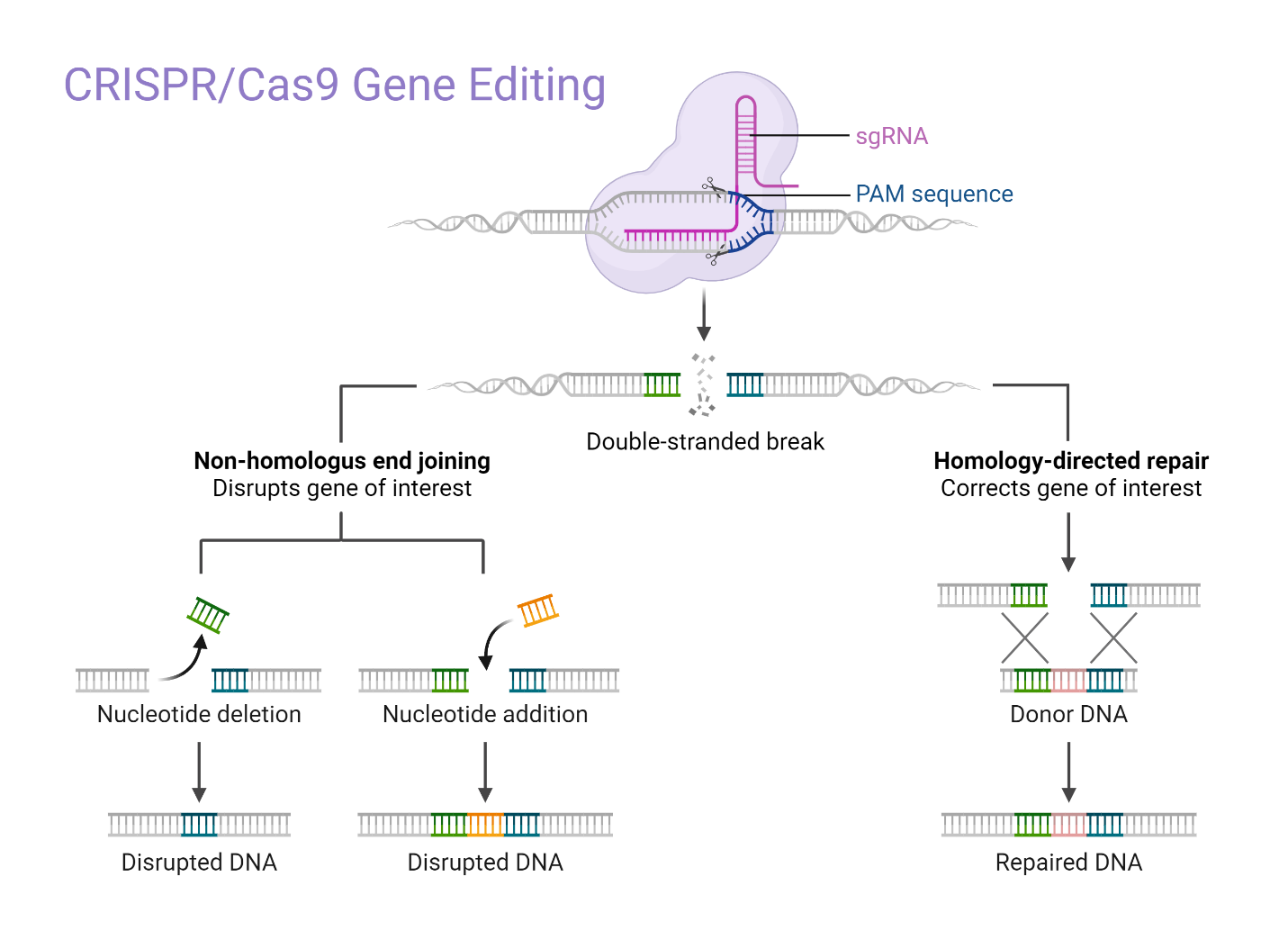
**

**Supplementary Figure 2:** Supplementary Figure: Mechanisms of CRISPR/Cas9-mediated Gene Editing. The diagram illustrates the targeted DNA cleavage by the CRISPR/Cas9 system, which can be followed by one of two DNA repair pathways. The Non-Homologous End Joining (NHEJ) pathway can lead to gene disruption via the introduction of nucleotide insertions or deletions, resulting in frameshift mutations. Alternatively, the Homology-Directed Repair (HDR) pathway utilizes a donor DNA template to achieve precise gene correction, restoring or altering the native DNA sequence [1].

**Supplementary Figure 3: Gene editing design for the six targets prioritized by the computational protocol**

**
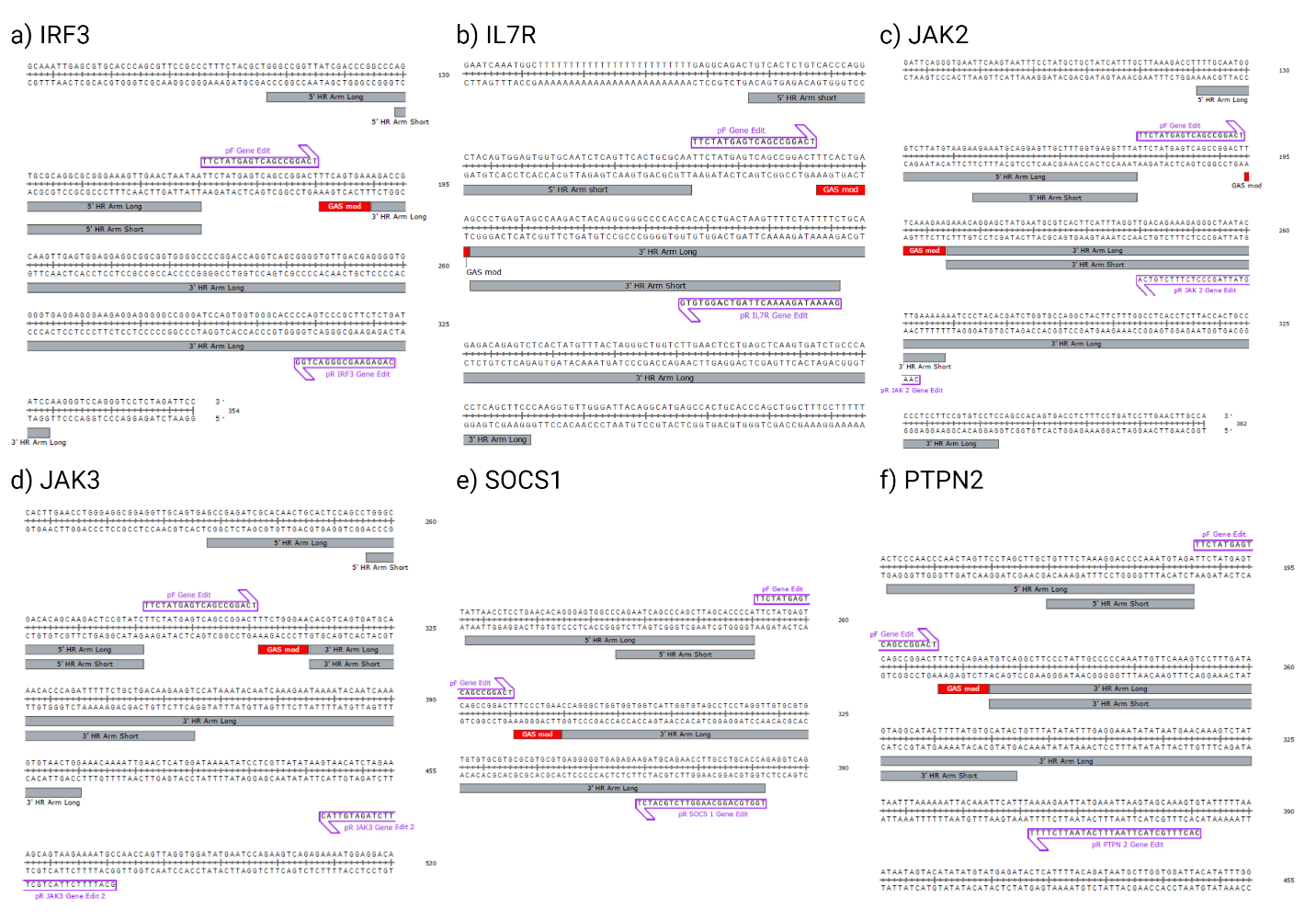
**

**Supplementary Figure 3:** Design of the gene editing machinery for the genes (a) IRF3, (b) IL7R, (c) JAK2, (d) JAK3, (e) SOCS1, and (f) PTPTN2, including their primer forwards (pF) and primer reverse (pR). We can use homology-directed repair for all of those targets using the following guide RNAs:

(a) IRF3
 <https://genome.ucsc.edu/cgi-bin/hgc?hgsid=1912842064_UOM8vyLUWBBcbHjfidEkDOfmvaWg&db=hg38&c=chr19&l=49665993&r=49666037&o=49665998&t=49666021&g=crisprAllTargets&i=>

(b) IL7R
<https://genome.ucsc.edu/cgi-bin/hgc?hgsid=1805717426_JErAu9qY0V2mVKIAQ2mbzWagtgFo&db=hg38&c=chr5&l=35853814&r=35853855&o=35853803&t=35853826&g=crisprAllTargets&i=>

(c) JAK2
<https://genome.ucsc.edu/cgi-bin/hgc?hgsid=1912460452_So31SOt4HGXnyLREraiYHnXPpm1S&db=hg38&c=chr9&l=4983684&r=4983753&o=4983738&t=4983761&g=crisprAllTargets&i=>

(d) JAK3
<https://genome.ucsc.edu/cgi-bin/hgc?hgsid=1912460452_So31SOt4HGXnyLREraiYHnXPpm1S&db=hg38&c=chr19&l=17851215&r=17851259&o=17851252&t=17851275&g=crisprAllTargets&i=>

(e) SOCS1
<https://genome.ucsc.edu/cgi-bin/hgc?hgsid=1912460452_So31SOt4HGXnyLREraiYHnXPpm1S&db=hg38&c=chr16&l=11257609&r=11257643&o=11257617&t=11257640&g=crisprAllTargets&i=>

(f) PTPN2
<https://genome.ucsc.edu/cgi-bin/hgc?hgsid=1912460452_So31SOt4HGXnyLREraiYHnXPpm1S&db=hg38&c=chr18&l=12886025&r=12886085&o=12886037&t=12886060&g=crisprAllTargets&i=>

Off-target considerations

(a) IRF3
guideRNA cuts in the intron area of BCL2L12. Check for BCL2L12 changes.

(g) SOCS1:

...*......*.........

chr17:73984197-73984219

(f) PTPN2:

Off-target effects

....T....A.......... AGG intergenic AC062032.1-RNU6-692P chr2:146946846 (+)

G.....A............. TGA intergenic PARD3-Y_RNA chr10:34159607 (+)

**Supplementary Figure 4: Visualization of IL4 stimulated ChIP-seq data for the six manually selected SNPs. (GSE100889)**

**
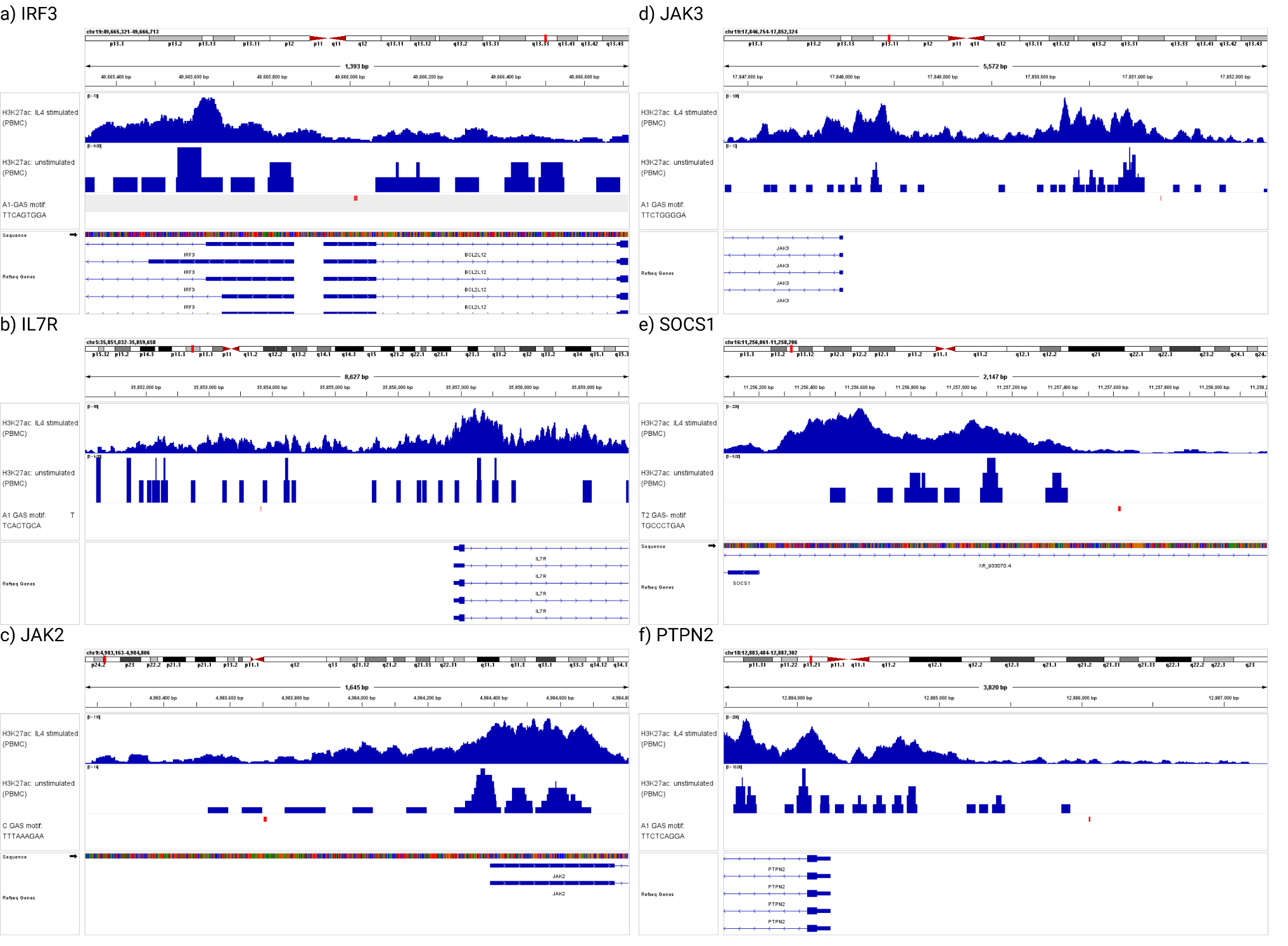
**

**Supplementary Table 1: A literature review on SNPs that can create or enhance motif activity in enhancers and promoters**

| **Organism** | **Disease/phenotype**  **associated** | **Transcription factor** | **Target gene** | **Short description of motif that enhances activity in enhancer or promoter** | **Citation** |
| --- | --- | --- | --- | --- | --- |
| Human | Prostate Cancer | HOXB13 | RFX6 | rs339331 alters the binding affinity of the transcription factor HOXB13 and induces the expression of RFX6. Multiple experiments for validation. | [2] |
| Human | Prostate Cancer | TCF4 (with the help of CTNNB1) | MYC | rs6983267 shows signs of an enhancing distant enhancer for MYC (59kb) in vivo using BACs. | [3,4] |
| Human | Hereditary Persistence of Fetal Hemoglobin (HPFH) | KLF1 | γ-globin | KLF1 binds to and activates the −198T>C γ-globin promoter in vitro and is potentially responsible for the persistent expression of γ-globin into adulthood. | [5] |
| Human | Sickle Cell Disease | GATA1 | γ-globin | −113A>G HPFH mutation does not disrupt BCL11A binding (repressor) but creates a de novo binding site for the transcriptional activator GATA1 | [6] |
| Human | HPFH | TAL1 | γ-globin | -175T>C point mutation associated with elevated fetal g-globin by creating TAL1 promoter binding. | [7] |
| Human | Multiple Sclerosis and Celiac Disease | GATA2 | HLA-F | rs2523393 creates a GATA2 binding site and upregulated HLA-F expression. | [8] |
| Human | Esophageal Cancer | c-MYB | COX-2 | −1195G→A change creates a c-MYB binding site and displays a higher promoter activity. | [9] |
| Human | Cervical Cancer | --- | APE1 | APE1 -656T>G increased the transcriptional activity of the APE1 gene, which could help to reduce risk of developing Cervical cancer. | [10] |
| Human | Hepatocellular Carcinoma | SP1 | MICA | rs2596538 strengthens the binding of SP1 and enhances the expression of MICA. | [11] |
| Human | Behçet’s Disease | TBX1 | IL-10 | rs3024490 within super-enhancer elements is able to specifically bind TBX1 and, in turn, promotes the enhancer activity and increased expression of IL-10 | [12] |
| Human | Drug Metabolism | --- | CYP2D6 | rs5758550 has been associated with enhanced transcription and suggested to be a useful marker of CYP2D6 activity. The authors concluded, while a minor expression change was observed, not to include this SNP in further trials. | [13] |
| Human | --- | --- | α-globin | SNP creates new promoter. | [14] |
| Human | Rheumatoid Arthritis and Type-2 Diabetes | PU.1 | TLR4 | rs7873784 in the 3′-untranslated region (3′-UTR) of the TLR4 gene enhances its expression in monocytes. | [15] |
| Human | Inflammatory Bowel Disease | --- | PXR1 | Several SNPs upstream of the PXR1 genes are associated with regulating the expression. | [16,17] |

**Supplementary Table 2: Number of motif candidates and SNP candidates after each step**

|  | Step 1 | Step 2 | Step 3 | Step 4 | Step 5 | Step 6 | Step 7 | Step 8 |
| --- | --- | --- | --- | --- | --- | --- | --- | --- |
| Candidates (motifs) | 17,039,967 | --- | --- | --- | --- | --- | --- | --- |
| Candidates (SNPs) | 3,301,512 | 50,265 | 16,017 | 4,391 | 416 | 273 | 214 | 30 / 6 |

**Step 1 = FIMO
Step 2 = H3K27ac
Step 3 = Immune and JAK-STAT pathway Gene Ontology genes
Step 4 = 10 kb upstream region
Step 5 = SNP found in dbSNP in at least two individuals
Step 6 = spacing of GAS motifs (at least 200 bps from each other)
Step 7 = same target available in the mouse genome
Step 8 = manual inspection and selection**

**Supplementary Table 3: ClinVar significance scores for selected SNPs reported by the data-driven projection before manual selection**

| **rsID** | **Gene** | **Significance** |
| --- | --- | --- |
| rs560898780 | IRF3 | N/A |
| rs762389787 | CSF3R | N/A |
| rs1209949613 | CD101 | N/A |
| rs566449782 | RORC | N/A |
| rs762957571 | ARHGEF2 | N/A |
| rs1320629886 | LBR | N/A |
| rs1257658099 | IL7R | N/A |
| rs1412706856 | ERBIN | N/A |
| rs556295198 | ERBIN | N/A |
| rs1166900155 | GCNT4 | N/A |
| rs538911235 | MSH3 | N/A |
| rs1211652644 | SLC12A2 | N/A |
| rs1300469777 | SLC12A2 | N/A |
| rs1244482467 | SLC12A2 | N/A |
| rs908390275 | TCF7 | N/A |
| rs1313579135 | TCF7 | N/A |
| rs1407404362 | TCF7 | N/A |
| rs1485439074 | ANKHD1 | N/A |
| rs898478536 | ITK | N/A |
| rs770307514 | ITK | N/A |
| rs1340591740 | IL12B | N/A |
| rs1054504837 | DOCK2 | N/A |
| rs539020058 | PRR7 | N/A |
| rs1328443888 | DUSP22 | N/A |
| rs964783222 | WRNIP1 | N/A |
| rs1290995737 | TMEM14C | N/A |
| rs1453228287 | TMEM14C | N/A |
| rs148371811 | JARID2 | N/A |
| rs1356982733 | GNL1 | N/A |
| rs1480616251 | DHX16 | N/A |
| rs1460296291 | TAPBP | N/A |
| rs940616305 | CGAS | N/A |
| rs1403816118 | FYN | N/A |
| rs1484558508 | VNN1 | N/A |
| rs56273545 | IFNGR1 | N/A |
| rs191964705 | IFNGR1 | N/A |
| rs999557026 | IFNGR1 | N/A |
| rs1243423407 | CITED2 | N/A |
| rs889130510 | CITED2 | N/A |
| rs1208491606 | EZR | N/A |
| rs1218894838 | PMS2 | N/A |
| rs926571814 | RAC1 | N/A |
| rs1286087613 | AHR | N/A |
| rs1328762768 | SKAP2 | N/A |
| rs1273816947 | ANLN | N/A |
| rs17496067 | CDK13 | N/A |
| rs891335123 | DBNL | N/A |
| rs891975351 | DBNL | N/A |
| rs562693929 | LAT2 | N/A |
| rs1242612897 | SLC25A40 | N/A |
| rs1339419515 | BPGM | N/A |
| rs1341587730 | BPGM | N/A |
| rs528194510 | CNOT4 | N/A |
| rs1262912023 | KIF13B | N/A |
| rs1328504999 | ASH2L | N/A |
| rs1023605113 | RIPK2 | N/A |
| rs1185793126 | RPL30 | N/A |
| rs1049184903 | RPL30 | N/A |
| rs146201851 | KLF10 | N/A |
| rs370669851 | JAK2 | N/A |
| rs1047339704 | CD274 | N/A |
| rs141486767 | CD274 | N/A |
| rs559384562 | CD274 | N/A |
| rs1389497816 | RRAGA | N/A |
| rs570659761 | C9orf72 | N/A |
| rs1340280311 | RIGI | N/A |
| rs183974004 | SIT1 | N/A |
| rs1164988424 | TRIM14 | N/A |
| rs552879920 | SLC46A2 | N/A |
| rs527767092 | SLC46A2 | N/A |
| rs760432279 | SLC46A2 | N/A |
| rs1166368265 | TRIM32 | N/A |
| rs1343459382 | ABL1 | N/A |
| rs1408230109 | VIM | N/A |
| rs1318433111 | VIM | N/A |
| rs1354909126 | ITGB1 | N/A |
| rs1288081410 | SHLD2 | N/A |
| rs1343658469 | C12orf4 | N/A |
| rs1156445361 | C12orf4 | N/A |
| rs537252651 | CD27 | N/A |
| rs746108614 | PHB2 | N/A |
| rs782801340 | C1RL | N/A |
| rs1235159557 | OLR1 | N/A |
| rs1353819412 | LRRK2 | N/A |
| rs369581626 | LMBR1L | N/A |
| rs372908103 | ZNF385A | N/A |
| rs1052974951 | TESPA1 | N/A |
| rs548115646 | RAB5B | N/A |
| rs11176083 | IRAK3 | N/A |
| rs889545139 | IRAK3 | N/A |
| rs1274171486 | LYZ | N/A |
| rs969252028 | EIF2B1 | N/A |
| rs1162946510 | RB1 | N/A |
| rs1489272927 | TRIM13 | N/A |
| rs934803990 | GPR183 | N/A |
| rs1234876633 | GPR183 | N/A |
| rs1333515728 | ANG | N/A |
| rs1320083106 | RNASE3 | N/A |
| rs1323186370 | RNASE3 | N/A |
| rs138171691 | RNASE3 | N/A |
| rs1016224188 | CEBPE | N/A |
| rs1023572517 | CNIH1 | N/A |
| rs1236319671 | GCH1 | N/A |
| rs1298660313 | ZFP36L1 | N/A |
| rs917109425 | BATF | N/A |
| rs1210326020 | YY1 | N/A |
| rs1225476024 | RCOR1 | N/A |
| rs570008008 | KLF13 | N/A |
| rs1298690347 | TYRO3 | N/A |
| rs1483085124 | PPIB | N/A |
| rs1263545453 | CSK | N/A |
| rs74025847 | CSK | N/A |
| rs957501412 | ISG20 | N/A |
| rs541463494 | LRRK1 | N/A |
| rs565093000 | MEFV | N/A |
| rs910130021 | SOCS1 | N/A |
| rs1285344328 | IL4R | N/A |
| rs139142497 | IL4R | N/A |
| rs1007157254 | LAT | N/A |
| rs1232784476 | MYL11 | N/A |
| rs965766271 | NOD2 | N/A |
| rs1173850428 | TRADD | N/A |
| rs938025290 | PSMB10 | N/A |
| rs1465699524 | PSMB10 | N/A |
| rs970365552 | EXOSC6 | N/A |
| rs1426850182 | RNF166 | N/A |
| rs1211118328 | RNF166 | N/A |
| rs566682313 | CXCL16 | N/A |
| rs1227693178 | C1QBP | N/A |
| rs1377617251 | EIF5A | N/A |
| rs917179073 | GPS2 | N/A |
| rs1015657660 | GPS2 | N/A |
| rs771380306 | VAMP2 | N/A |
| rs1427979634 | PIK3R5 | N/A |
| rs747210934 | SUPT6H | N/A |
| rs73285000 | RFFL | N/A |
| rs981669204 | RFFL | N/A |
| rs565692092 | NR1D1 | N/A |
| rs1428746001 | RARA | N/A |
| rs1291868776 | TMEM106A | N/A |
| rs574137262 | HEXIM1 | N/A |
| rs898372560 | SP2 | N/A |
| rs34167845 | CD300A | N/A |
| rs1262896680 | CD300E | N/A |
| rs1239109578 | UNC13D | N/A |
| rs567920860 | SPHK1 | N/A |
| rs181657872 | JMJD6 | N/A |
| rs912159055 | ACTG1 | N/A |
| rs949644659 | ACTG1 | N/A |
| rs1315780565 | COLEC12 | N/A |
| rs138606888 | PTPN2 | N/A |
| rs932943623 | MKNK2 | N/A |
| rs113402573 | SPPL2B | N/A |
| rs1389583407 | VAV1 | N/A |
| rs144739782 | TMED1 | N/A |
| rs540787753 | ACP5 | N/A |
| rs990431105 | KLF2 | N/A |
| rs952750220 | KLF2 | N/A |
| rs571421696 | JAK3 | N/A |
| rs143632289 | FFAR2 | N/A |
| rs1268829036 | FFAR2 | N/A |
| rs1327438586 | BCL3 | N/A |
| rs948542112 | CLPTM1 | N/A |
| rs1029399914 | BRD1 | N/A |
| rs1209008068 | BID | N/A |
| rs1327557545 | KLRB1 | N/A |
| rs1164186254 | KLRB1 | N/A |
| rs1207563094 | TNFRSF13C | N/A |
| rs1415066403 | CCDC134 | N/A |
| rs1416479337 | RHBDD3 | N/A |
| rs1313866100 | LGALS1 | N/A |
| rs953877230 | GTPBP1 | N/A |
| rs545618650 | GTPBP1 | N/A |
| rs39519520 | ATF4 | N/A |
| rs1010340846 | CCDC134 | N/A |
| rs1415066403 | CCDC134 | N/A |
| rs1207563094 | TNFRSF13C | N/A |

**Supplementary Table 4: RNA-seq samples**

1. **GSE215771; A549 cell line stimulated with IFNg (RNA-seq)**

|  | DMSO | DMSO + IFNg |
| --- | --- | --- |
| Sample numbers | 3 | 3 |

1. **GSE178640; A549 cell line stimulated with IFNa (RNA-seq)**

|  | DMSO | DMSO + IFNa |
| --- | --- | --- |
| Sample numbers | 3 | 3 |

1. **GSE35267; A549 cell line stimulated with IFNb (RNA-seq)**

|  | Untreated | Untreated + IFNb |
| --- | --- | --- |
| Sample numbers | 3 | 3 |

1. **GSE46599; JURKAT cell line stimulated with IFNb (NanoString)**

|  | Untreated | Untreated + IFNb |
| --- | --- | --- |
| Sample numbers | 2 | 2 |

**Supplementary Table 5: Primers for Potential Experiments (analyzed using IDT oligo analyzer** [**https://www.idtdna.com/pages/tools/oligoanalyzer?utm_source=google&utm_medium=cpc&utm_campaign=00583_1a_03&utm_content=search&gad_source=1&gclid=CjwKCAjwp4m0BhBAEiwAsdc4aBV4TPU66PLtgFi3nCBqOAUrX0Zinc8tEKQ1AwxClnfIj8hRhp-RchoCqqcQAvD_BwE**](https://www.idtdna.com/pages/tools/oligoanalyzer?utm_source=google&utm_medium=cpc&utm_campaign=00583_1a_03&utm_content=search&gad_source=1&gclid=CjwKCAjwp4m0BhBAEiwAsdc4aBV4TPU66PLtgFi3nCBqOAUrX0Zinc8tEKQ1AwxClnfIj8hRhp-RchoCqqcQAvD_BwE)**)**

| Gene | Amplicon primers | Primers for measuring gene expression (e.g., RT-qPCR) |
| --- | --- | --- |
| IRF3 | pF (5’-3’):  TTCTATGAGTCAGCCGGACT  rF (5’-3’):  CCAGTCCCGCTTCTCTG | pF (5’-3’):  TCTGCCCTCAACCGCAAAGAAG  rF (5’-3’):  TACTGCCTCCACCATTGGTGTC |
| IL7R | pF (5’-3’):  TTCTATGAGTCAGCCGGACT  rF (5’-3’):  CACACCTGACTAAGTTTTCTATTTTC | pF (5’-3’):  ATCGCAGCACTCACTGACCTGT  rF (5’-3’):  TCAGGCACTTTACCTCCACGAG |
| JAK2 | pF (5’-3’):  TTCTATGAGTCAGCCGGACT  rF (5’-3’):  TGACAGAAAGAGGGCTAATACTTG | pF (5’-3’):  CCAGATGGAAACTGTTCGCTCAG  rF (5’-3’):  GAGGTTGGTACATCAGAAACACC |
| JAK3 | pF (5’-3’):  TTCTATGAGTCAGCCGGACT  rF (5’-3’):  GTAACATCTAGAAAGCAGTAAGAAAATGC | pF (5’-3’):  AGTGACCCTCACTTCCTGCTGT  rF (5’-3’):  GGCTGAACCAAGGATGATGTGG |
| SOCS1 | pF (5’-3’):  TTCTATGAGTCAGCCGGACT  rF (5’-3’):  AGATGCAGAACCTTGCCTGCACCA | pF (5’-3’):  TTCGCCCTTAGCGTGAAGATGG  rF (5’-3’):  TAGTGCTCCAGCAGCTCGAAGA |
| PTPN2 | pF (5’-3’):  TTCTATGAGTCAGCCGGACT  rF (5’-3’):  AAAAGAATTATGAAATTAAGTAGCAAAGTG | pF (5’-3’):  CATGCTGAACCGCATTGTGGAG  rF (5’-3’):  GACAAGAGCTTCACACTGAATCC |

**Supplementary Text 1: Summary of literature research articles**

Huang et al. discovered that the prostate cancer risk-associated SNP rs339331 at 6q22 enhances the binding of HOXB13 to a transcriptional enhancer, leading to allele-specific upregulation of the gene RFX6. Suppression of RFX6 was found to diminish prostate cancer cell proliferation, migration, and invasion, highlighting its potential role in tumor progression. This association between the SNP and increased RFX6 mRNA levels in prostate tumors, along with clinical data indicating that RFX6 upregulation correlates with tumor progression and metastasis, suggests that rs339331 affects prostate cancer risk by altering RFX6 expression [2]. Wasserman et al. focused on the association between noncoding variants at the 8q24 locus and the risk of various cancers, including prostate cancer. They identified the rs6983267 SNP within a prostate enhancer active in vivo, which is also associated with colorectal and breast cancers. This SNP alters enhancer activity, increasing the expression of the nearby MYC proto-oncogene in prostate tissues. The risk allele for rs6983267 increases prostate enhancer activity, suggesting that early alterations in MYC expression mediated by this SNP may influence cancer risk [3]. Sotelo et al. demonstrated that the 8q24 locus, known for its association with increased cancer risk, contains multiple enhancer elements capable of regulating the transcription of the MYC gene. They showed that one specific enhancer interacts with the MYC promoter through the TF TCF-4 and operates in an allele-specific manner. This finding suggests that genetic variants within these enhancers, such as SNP rs6983267, could influence MYC expression levels, potentially impacting cancer susceptibility [4]. Wienert et al. identified a mutation (2198T>C) in the fetal globin gene promoter that significantly elevates fetal hemoglobin (HbF) expression, a beneficial trait in individuals with beta-hemoglobinopathies. This mutation creates a de novo binding site for Krueppel-Like Factor 1 (KLF1), a potent erythroid activator, resulting in elevated HbF levels. The study's CRISPR-mediated introduction of the 2198T>C mutation into human erythroid cells and subsequent analyses underline the potential of targeting such naturally occurring mutations to therapeutically increase HbF levels in diseases like sickle cell anemia and beta-thalassemia [5]. Martyn et al. identified a regulatory mutation (2113A>G) that creates a de novo GATA1 binding site in the fetal globin promoter, elevating fetal hemoglobin levels without affecting BCL11A binding [6]. Wienert et al. introduced a beneficial naturally occurring mutation (−175 T>C) associated with elevated fetal hemoglobin levels into erythroid cell lines. This mutation creates a de novo binding site for the TF TAL1, promoting chromatin looping from distal enhancers to the γ-globin promoter, thereby increasing fetal globin expression [7]. The study by Mika et al. investigates a G/A polymorphism at SNP rs2523393, which significantly influences fecundability—the probability of becoming pregnant within a single menstrual cycle—by affecting HLA-F expression. The A allele of rs2523393 creates a GATA2 binding site within a progesterone-responsive distal enhancer that loops to the HLA-F promoter [8]. Zhang et al. explored the functional genetic variants in the cyclooxygenase-2 (COX-2) promoter and their link to esophageal cancer risk. They identified three SNPs (-1290A>G, -1195G>A, -765G>C), with the -1195G>A variant creating a c-MYB binding site, leading to higher promoter activity. Particularly, the -1195A allele was associated with significantly increased risks for esophageal squamous cell carcinoma (ESCC), suggesting genetic variations in COX-2 could influence individual susceptibility to esophageal cancer [9]. Wang et al. demonstrated that the APE1 -656T>G polymorphism in the promoter region is associated with a reduced risk of cervical cancer. The polymorphism enhances the binding affinity of transcriptional factors, leading to increased APE1 expression [10]. Lo et al. identified a functional variant, SNP rs2596538, in the MICA promoter that affects the binding of TF SP1 and influences the risk of hepatitis C virus (HCV)-related hepatocellular carcinoma (HCC). The G allele of rs2596538 enhances SP1 binding and is associated with higher MICA expression and increased risk for HCV-related HCC [11]. Tan et al. explored how a specific noncoding SNP, rs3024490, within super-enhancer elements can influence IL-10 expression and the risk of Behçet’s disease. The T allele of rs3024490 is found to specifically bind the transcription factor TBX1, which enhances the enhancer activity and leads to increased IL-10 expression. This mechanism suggests a protective role against Behçet’s disease, attributed to the anti-inflammatory effects of IL-10 [12]. Dinh et al. assessed the influence of the CYP2D6 "enhancer" SNP, rs5758550, on enzyme activity, concluding its impact was minimal or inconclusive across studies. Despite initial suggestions that rs5758550 might enhance CYP2D6 transcription, incorporating this SNP into models did not significantly improve the prediction of enzyme activity beyond what is predicted by the CYP2D6 genotype alone​ [13]. Bozhilov et al. demonstrated how a single nucleotide variant (SNV) within the human α-globin cluster creates a new promoter and an associated unidirectional transcript. This SNV, causing a T to C transition, downregulates α-globin expression and contributes to α-thalassemia. The new promoter disrupts the interaction between α-globin genes and their associated super-enhancer in an orientation-dependent manner [14]. Korneev et al. investigated the SNP rs7873784 in the 3′-untranslated region (3′-UTR) of the TLR4 gene, which is associated with rheumatoid arthritis and type-2 diabetes mellitus. They discovered that the minor C allele of this SNP creates a binding site for the transcription factor PU.1, enhancing TLR4 expression in monocytes. This increased expression, driven by the PU.1 transcription factor's binding, could modulate the development of pathologies involving chronic inflammation [15]. Zhang et al. explored the human pregnane X receptor (PXR), which activates the CYP3A4 gene crucial for drug metabolism. They identified 38 SNPs affecting PXR function and gene expression [16]. Rana et al. (2017) identified a critical single nucleotide polymorphism (SNP) in the promoter of the PXR gene, which plays a key role in the metabolism and detoxification of substances. The SNP, located at position -831 from the transcriptional start site (rs3814055), switches between a C and a T allele. They found that the T-allele was associated with significantly greater transcriptional activity than the C-allele, indicating a direct effect on the transcriptional regulation of the PXR gene [17].

**Supplementary Text 2: Potential future directions**

We selected the JURKAT cell line (i.e., a human T lymphocyte cell line derived from a patient with acute T cell leukemia) and the A549 cell line (i.e., a human lung carcinoma cell line) given their role in immune response [18] and vulnerability of lung tissues to infectious diseases [19]. We aimed to assess if the critical components of the JAK-STAT pathway (i.e., JAK1-3, STAT1-6) and the target genes are expressed in the cell lines and if they are under the control of the JAK-STAT pathway by searching for stimulated cell lines in the Gene Expression Omnibus (GEO) database [20]. In order to screen the functional impact of the identified SNPs within the JAK-STAT pathway enhancers, we propose a luciferase reporter assay in which either the wild-type (as a control) or the different variants containing the SNP are cloned upstream of a luciferase reporter gene in an expression plasmid. This method has been extensively validated for assessing gene expression modulation by transcriptional enhancers, allowing to quantify enhancer activity [21]. Next, we need to investigate how we could transfect these cells effectively with the genome-editing machinery by conducting literature research on them. For JURKAT, the prevalent method for transfection is electroporation (i.e., brief electrical pulse to create temporary pores in the cell membrane, allowing molecular entry) [22–24], while for A549 cells, lipofectamine (i.e., by encapsulating the gene editing machinery into lipid nanoparticles, which then merge with the cell membrane) seems to be an often employed method [25,26]. The target cell lines are then transfected with these plasmids, and the impact of the SNP-derived enhancers is assessed by comparing luciferase activity in cell lysates with the wild-type control [21]. We want to point out that the luciferase reporter assay is viable for seeking preliminary evidence that our hypotheses could be true. However, we strongly believe that the genome must be stably edited to elucidate its function.

Following this initial activity screening, we need to decide which genome-editing machinery could be used to introduce the SNP into the genome. CRISPR/Cas9, a genome editing tool, allows for precise DNA alterations by creating double-strand breaks at targeted locations using guideRNAs (gRNAs, i.e., RNAs that can guide the CRISPR/Cas9 complex to the target site), which are then repaired by the cell [27,28]. The current approaches that enable precise editing of a single nucleotide in the target sequence are Base Editors and Prime Editing. Base Editors combine a deactivated Cas9 with a deaminase enzyme, enabling direct conversion of DNA bases without double-strand breaks, offering higher precision but limited to certain base changes [29]. Prime Editing [30] merges the Cas9 endonuclease with a reverse transcriptase, allowing for a wider range of edits, including insertions, deletions, and substitutions, without double-strand breaks [31–33] This provides broader applicability but with varying efficiency across cell types [31–33]. Considering our specific target sequences, we could not identify suitable PAM sites (NGG) or guide RNAs to enable the use of base- or prime editors. Thus, we pursued a Homology-Directed Repair (HDR, Suppl. Fig. 2) approach by designing a set of gRNAs to target each of the locations and a donor sequence to be integrated at the double-strand break site, generating the SNP. Guide RNAs were designed following the standard guidelines to maximize efficiency and specificity. A good guideRNAs is 18-25 base pairs long and has no 0- or 1-mismatches throughout the genome; 2-mismatches throughout the genome are okay if both mismatches throughout the genome are in the seed region (i.e., the last 10 bases in 5' to 3' direction); 3- or 4-mismatches should have at least 2 mismatches in the seed region; this approach prevents off-target effects of the CRISPR/Cas9 complex. Once the CRISPR/Cas9 complex breaks both strands of the DNA, the repair mechanisms of the cell are triggered [34–36]. The donor sequence design consists of the SNP, flanked by sequence-specific homology and an artificial primer-binding site (Suppl. Fig. 3). This design ensures the destruction of the PAM site to avoid additional cuts in the modified cells. After establishment and screening of modified cells, the effect of the SNPs on gene expression will be evaluated by qPCR (Suppl. Table 5) [1].

Next, we need to identify the cells that were successfully transfected. We could do this by including a promoter and the sequence for the green fluorescent protein (GFP), which would turn the cell green after a while. However, inserting such a long sequence could alter the desired enhancer site. Alternatively, we could use a CRISPR/Cas9 complex, which is coupled to a GFP. The advantage is that the GFP is only temporarily in the cell. This enables fluorescence-activated cell sorting (FACS) to isolate single-cell clones that were effectively transfected. However, as outlined above and in Suppl. Fig. 1, just because the CRISPR/Cas9 complex coupled with GFP is inside the cell doesn’t guarantee that the HDR was successful, so we need to execute an additional technique to identify the cells, which is called single-cell cloning (i.e., putting a single cell into a well and let it divide until a sufficient number of cells are in the well to establish a colony). Once sufficient cells are grown from the single cells, we need to distinguish which cells have the correct mutation without off-target effects or deletions/insertions in front or behind the mutated site. This is enabled by detecting the presence of a short amplicon amplified by a forward primer targeting the unique artificial primer-binding site introduced in the donor HDR template and a reverse primer specific to each of the genes of interest.

After establishing the modified cell lines, we can perform different assays to investigate the role of the SNPs, namely, ChIP-seq, RNA-seq, and/or surface assays. Moreover, cells can be treated with interferons or cytokines to activate the JAK-STAT pathway [37].

**Supplementary Text 3: Limitations of potential future experimental approaches**

While efficient, the use of a luciferase reporter assay for the rapid determination of enhancer activity carries the risk of false positives [38]. Additionally, the technical and financial challenges of incorporating a 10kb region into a luciferase reporter construct further complicate the experimental design. Gene editing techniques, integral to our approach, are constrained by factors such as the design of amplicons and spacers, and the potential for off-target effects cause problems with the interpretation of the mutations to a real-world scenario where such elements are not present.

The transfection of the CRISPR/Cas9 cells is inherently stressful, and the subsequent FACS adds further stress, which could harm the cells. Some of the cells may not survive this compounded stress, an issue that is particularly pertinent given the coupling of GFP with the CRISPR/Cas9 complex for the identification of successfully transfected cells. While facilitating the identification process, this method does not ensure the desired outcome of HDR. Moreover, the process of single-cell cloning, essential for establishing cell lines with the desired genetic modifications, introduces additional challenges. Cells are typically accustomed to a communal environment, and isolation can lead to stress and, consequently, higher mortality rates among cloned cells.

**Supplementary Textbox 1: Canonical and non-canonical GAS motif input for FIMO of the MEME suite**

Background letter frequencies (from unknown source):

A 0.250 C 0.250 G 0.250 T 0.250

MOTIF 1 TTCNNNGAA

letter-probability matrix: alength= 4 w= 9 nsites= 1 E= 0e+0

0.000000 0.000000 0.000000 1.000000

0.000000 0.000000 0.000000 1.000000

0.000000 1.000000 0.000000 0.000000

0.250000 0.250000 0.250000 0.250000

0.250000 0.250000 0.250000 0.250000

0.250000 0.250000 0.250000 0.250000

0.000000 0.000000 1.000000 0.000000

1.000000 0.000000 0.000000 0.000000

1.000000 0.000000 0.000000 0.000000

MOTIF 2 TTCNNNNGAA

letter-probability matrix: alength= 4 w= 10 nsites= 1 E= 0e+0

0.000000 0.000000 0.000000 1.000000

0.000000 0.000000 0.000000 1.000000

0.000000 1.000000 0.000000 0.000000

0.250000 0.250000 0.250000 0.250000

0.250000 0.250000 0.250000 0.250000

0.250000 0.250000 0.250000 0.250000

0.250000 0.250000 0.250000 0.250000

0.000000 0.000000 1.000000 0.000000

1.000000 0.000000 0.000000 0.000000

1.000000 0.000000 0.000000 0.000000

**Supplementary Textbox 2: Potential almost GAS motifs for T1 GAS, T2 GAS, C GAS, G GAS, A1 GAS, and A2 GAS**

MEME version 4

ALPHABET= ACGT

strands: + -

Background letter frequencies (from unknown source):

A 0.250 C 0.250 G 0.250 T 0.250

MOTIF 1 VTCNNNGAA

letter-probability matrix: alength= 4 w= 9 nsites= 1 E= 0e+0

0.333333 0.333333 0.333333 0.000000

0.000000 0.000000 0.000000 1.000000

0.000000 1.000000 0.000000 0.000000

0.250000 0.250000 0.250000 0.250000

0.250000 0.250000 0.250000 0.250000

0.250000 0.250000 0.250000 0.250000

0.000000 0.000000 1.000000 0.000000

1.000000 0.000000 0.000000 0.000000

1.000000 0.000000 0.000000 0.000000

MOTIF 2 TVCNNNGAA

letter-probability matrix: alength= 4 w= 9 nsites= 1 E= 0e+0

0.000000 0.000000 0.000000 1.000000

0.333333 0.333333 0.333333 0.000000

0.000000 1.000000 0.000000 0.000000

0.250000 0.250000 0.250000 0.250000

0.250000 0.250000 0.250000 0.250000

0.250000 0.250000 0.250000 0.250000

0.000000 0.000000 1.000000 0.000000

1.000000 0.000000 0.000000 0.000000

1.000000 0.000000 0.000000 0.000000

MOTIF 3 TTDNNNGAA

letter-probability matrix: alength= 4 w= 9 nsites= 1 E= 0e+0

0.000000 0.000000 0.000000 1.000000

0.000000 0.000000 0.000000 1.000000

0.333333 0.000000 0.333333 0.333333

0.250000 0.250000 0.250000 0.250000

0.250000 0.250000 0.250000 0.250000

0.250000 0.250000 0.250000 0.250000

0.000000 0.000000 1.000000 0.000000

1.000000 0.000000 0.000000 0.000000

1.000000 0.000000 0.000000 0.000000

MOTIF 4 TTCNNNHAA

letter-probability matrix: alength= 4 w= 9 nsites= 1 E= 0e+0

0.000000 0.000000 0.000000 1.000000

0.000000 0.000000 0.000000 1.000000

0.000000 1.000000 0.000000 0.000000

0.250000 0.250000 0.250000 0.250000

0.250000 0.250000 0.250000 0.250000

0.250000 0.250000 0.250000 0.250000

0.333333 0.333333 0.000000 0.333333

1.000000 0.000000 0.000000 0.000000

1.000000 0.000000 0.000000 0.000000

MOTIF 5 TTCNNNGBA

letter-probability matrix: alength= 4 w= 9 nsites= 1 E= 0e+0

0.000000 0.000000 0.000000 1.000000

0.000000 0.000000 0.000000 1.000000

0.000000 1.000000 0.000000 0.000000

0.250000 0.250000 0.250000 0.250000

0.250000 0.250000 0.250000 0.250000

0.250000 0.250000 0.250000 0.250000

0.000000 0.000000 1.000000 0.000000

0.000000 0.333333 0.333333 0.333333

1.000000 0.000000 0.000000 0.000000

MOTIF 6 TTCNNNGAB

letter-probability matrix: alength= 4 w= 9 nsites= 1 E= 0e+0

0.000000 0.000000 0.000000 1.000000

0.000000 0.000000 0.000000 1.000000

0.000000 1.000000 0.000000 0.000000

0.250000 0.250000 0.250000 0.250000

0.250000 0.250000 0.250000 0.250000

0.250000 0.250000 0.250000 0.250000

0.000000 0.000000 1.000000 0.000000

1.000000 0.000000 0.000000 0.000000

0.000000 0.333333 0.333333 0.333333

**References**

1. Sansbury BM, Kmiec EB. On the origins of homology directed repair in mammalian cells. Int J Mol Sci. 2021;22: 3348.

2. Huang Q, Whitington T, Gao P, Lindberg JF, Yang Y, Sun J, et al. A prostate cancer susceptibility allele at 6q22 increases RFX6 expression by modulating HOXB13 chromatin binding. Nat Genet. 2014;46: 126–135.

3. Wasserman NF, Aneas I, Nobrega MA. An 8q24 gene desert variant associated with prostate cancer risk confers differential in vivo activity to a *MYC* enhancer. Genome Res. 2010;20: 1191–1197.

4. Sotelo J, Esposito D, Duhagon MA, Banfield K, Mehalko J, Liao H, et al. Long-range enhancers on 8q24 regulate c-Myc. Proc Natl Acad Sci U S A. 2010;107: 3001–3005.

5. Wienert B, Martyn GE, Kurita R, Nakamura Y, Quinlan KGR, Crossley M. KLF1 drives the expression of fetal hemoglobin in British HPFH. Blood. 2017;130: 803–807.

6. Martyn GE, Wienert B, Kurita R, Nakamura Y, Quinlan KGR, Crossley M. A natural regulatory mutation in the proximal promoter elevates fetal globin expression by creating a de novo GATA1 site. Blood. 2019;133: 852–856.

7. Wienert B, Funnell APW, Norton LJ, Pearson RCM, Wilkinson-White LE, Lester K, et al. Editing the genome to introduce a beneficial naturally occurring mutation associated with increased fetal globin. Nat Commun. 2015;6: 1–8.

8. Mika KM, Li X, DeMayo FJ, Lynch VJ. An ancient fecundability-associated polymorphism creates a GATA2 binding site in a distal enhancer of HLA-F. Am J Hum Genet. 2018;103: 509–521.

9. Zhang X, Miao X, Tan W, Ning B, Liu Z, Hong Y, et al. Identification of functional genetic variants in and their association with risk of esophageal cancer. Gastroenterology. 2005;129: 565–576.

10. Wang M, Chu H, Wang S, Wang M, Wang W, Han S, et al. Genetic variant in APE1 gene promoter contributes to cervical cancer risk. Am J Obstet Gynecol. 2013;209: 360.e1-360.e7.

11. Lo PHY, Urabe Y, Kumar V, Tanikawa C, Koike K, Kato N, et al. Identification of a functional variant in the MICA promoter which regulates MICA expression and increases HCV-related hepatocellular carcinoma risk. PLoS One. 2013;8: e61279.

12. Tan H, Su G, Tan X, Qin Y, Chen L, Yuan G, et al. SNP-mediated binding of TBX1 to the enhancer element of*IL-10*reduces the risk of Behçet’s disease. Epigenomics. 2021;13: 1523–1537.

13. Dinh JC, Boone EC, Staggs VS, Pearce RE, Wang WY, Gaedigk R, et al. The impact of the CYP2D6 “enhancer” single nucleotide polymorphism on CYP2D6 activity. Clin Pharmacol Ther. 2022;111: 646–654.

14. Bozhilov YK, Downes DJ, Telenius J, Marieke Oudelaar A, Olivier EN, Mountford JC, et al. A gain-of-function single nucleotide variant creates a new promoter which acts as an orientation-dependent enhancer-blocker. Nat Commun. 2021;12: 1–13.

15. Korneev KV, Sviriaeva EN, Mitkin NA, Gorbacheva AM, Uvarova AN, Ustiugova AS, et al. Minor C allele of the SNP rs7873784 associated with rheumatoid arthritis and type-2 diabetes mellitus binds PU.1 and enhances TLR4 expression. Biochim Biophys Acta Mol Basis Dis. 2020;1866: 165626.

16. Zhang J, Kuehl P, Green ED, Touchman JW, Watkins PB, Daly A, et al. The human pregnane X receptor: genomic structure and identification and functional characterization of natural allelic variants. Pharmacogenet Genomics. 2001;11: 555.

17. Rana M, Coshic P, Goswami R, Tyagi RK. Influence of a critical single nucleotide polymorphism on nuclear receptor PXR‐promoter function. Cell Biol Int. 2017;41: 570–576.

18. Smith-Garvin JE, Koretzky GA, Jordan MS. T cell activation. T cell activation Annual Review of Immunology. 2009;27: 591–619.

19. Shaykhiev R, Crystal RG. Disease pathogenesis of lung infections. American Journal of Respiratory Cell and Molecular Biology. 2013;49: 875–880.

20. Clough E, Barrett T. The Gene Expression Omnibus Database. Methods Mol Biol. 2016;1418: 93–110.

21. Lin Y, Meng F, Fang C, Zhu B, Jiang J. Rapid validation of transcriptional enhancers using agrobacterium-mediated transient assay. Plant Methods. 2019;15. doi:10.1186/s13007-019-0407-y

22. De Silva D, Ferguson L, Chin GH, Smith BE, Apathy RA, Roth TL, et al. Robust T cell activation requires an eIF3-driven burst in T cell receptor translation. Elife. 2021;10: e74272.

23. Weng N, Miller M, Pham AK, Komor AC, Broide DH. Single‐base editing of rs12603332 on chromosome 17q21 with a cytosine base editor regulates ORMDL3 and ATF6α expression. Allergy. 2022;77: 1139–1149.

24. Gehl J. Electroporation: theory and methods, perspectives for drug delivery, gene therapy and research. Acta Physiol Scand. 2003;177: 437–447.

25. Asgari A, Lesyk G, Poitras E, Govindasamy N, Terry K, To R, et al. Platelets stimulate programmed death‐ligand 1 expression by cancer cells: Inhibition by anti‐platelet drugs. J Thromb Haemost. 2021;19: 2862–2872.

26. Van Royen T, Sedeyn K, Moschonas GD, Toussaint W, Vuylsteke M, Van Haver D, et al. An unexpected encounter: Respiratory syncytial virus nonstructural protein 1 interacts with mediator subunit MED25. J Virol. 2022;96. doi:10.1128/jvi.01297-22

27. Ishino Y, Shinagawa H, Makino K, Amemura M, Nakata A. Nucleotide sequence of the iap gene, responsible for alkaline phosphatase isozyme conversion in Escherichia coli, and identification of the gene product. J Bacteriol. 1987;169: 5429–5433.

28. Gostimskaya I. CRISPR–Cas9: A history of its discovery and ethical considerations of its use in genome editing. Biochemistry (Mosc). 2022;87: 777–788.

29. Rees HA, Liu DR. Base editing: precision chemistry on the genome and transcriptome of living cells. Nat Rev Genet. 2018;19: 770–788.

30. Lee HK, Willi M, Miller SM, Kim S, Liu C, Liu DR, et al. Targeting fidelity of adenine and cytosine base editors in mouse embryos. Nat Commun. 2018;9: 1–6.

31. Chen PJ, Liu DR. Prime editing for precise and highly versatile genome manipulation. Nat Rev Genet. 2023;24: 161–177.

32. Anzalone AV, Randolph PB, Davis JR, Sousa AA, Koblan LW, Levy JM, et al. Search-and-replace genome editing without double-strand breaks or donor DNA. Nature. 2019;576: 149–157.

33. Scholefield J, Harrison PT. Prime editing - an update on the field. Gene Ther. 2021;28: 396–401.

34. Matson AW, Hosny N, Swanson ZA, Hering BJ, Burlak C. Optimizing sgRNA length to improve target specificity and efficiency for the GGTA1 gene using the CRISPR/Cas9 gene editing system. PLoS One. 2019;14: e0226107.

35. Mohr SE, Hu Y, Ewen-Campen B, Housden BE, Viswanatha R, Perrimon N. CRISPR guide RNA design for research applications. FEBS J. 2016;283: 3232–3238.

36. Malik A, Gul A, Munir F, Amir R, Alipour H, Babar MM, et al. Evaluating the cleavage efficacy of CRISPR-Cas9 sgRNAs targeting ineffective regions of *Arabidopsis thaliana* genome. PeerJ. 2021;9: e11409.

37. Hu X, Li J, Fu M, Zhao X, Wang W. The JAK/STAT signaling pathway: from bench to clinic. Signal Transduct Target Ther. 2021;6: 402.

38. Yonchev D, Bajorath J. Inhibitor bias in luciferase-based luminescence assays. Future Sci OA. 2020;6. doi:10.2144/fsoa-2020-0081
